# The Effect of Methylselenocysteine and Sodium Selenite Treatment on microRNA Expression in Liver Cancer Cell Lines

**DOI:** 10.1007/s12253-020-00870-8

**Published:** 2020-07-12

**Authors:** Gábor Lendvai, Tímea Szekerczés, Endre Kontsek, Arun Selvam, Attila Szakos, Zsuzsa Schaff, Mikael Björnstedt, András Kiss

**Affiliations:** 1 2nd Department of Pathology, Semmelweis University, Ulloi 93, Budapest, H-1091 Hungary; 2 Division of Pathology, F46, Department of Laboratory Medicine, Karolinska Institutet, Karolinska University Hospital Huddinge, SE-14186 Stockholm, Sweden

**Keywords:** Methylselenocysteine, Sodium selenite, microRNA expression, Hepatocellular carcinoma, Cholangiocarcinoma, IC50 value

## Abstract

The unique character of selenium compounds, including sodium selenite and Se-methylselenocysteine (MSC), is that they exert cytotoxic effects on neoplastic cells, providing a great potential for treating cancer cells being highly resistant to cytostatic drugs. However, selenium treatment may affect microRNA (miRNA) expression as the pattern of circulating miRNAs changed in a placebo-controlled selenium supplement study. This necessitates exploring possible changes in the expression profiles of miRNAs. For this, miRNAs being critical for liver function were selected and their expression was measured in hepatocellular carcinoma (HLE and HLF) and cholangiocarcinoma cell lines (TFK-1 and HuH-28) using individual TaqMan MicroRNA Assays following selenite or MSC treatments. For establishing tolerable concentrations, IC_50_ values were determined by performing SRB proliferation assays. The results revealed much lower IC_50_ values for selenite (from 2.7 to 11.3 μM) compared to MSC (from 79.5 to 322.6 μM). The treatments resulted in cell line-dependent miRNA expression patterns, with all miRNAs found to show fold change differences; however, only a few of these changes were statistically different in treated cells compared to untreated cells below IC_50_. Namely, miR-199a in HLF, miR-143 in TFK-1 upon MSC treatment, miR-210 in HLF and TFK-1, miR-22, -24, -122, −143 in HLF upon selenite treatment. Fold change differences revealed that miR-122 with both selenium compounds, miR-199a with MSC and miR-22 with selenite were affected. The miRNAs showing minimal alterations included miR-125b and miR-194. In conclusion, our results revealed moderately altered miRNA expression in the cell lines (less alterations following MSC treatment), being miR-122, −199a the most affected and miR-125b, -194 the least altered miRNAs upon selenium treatment.

## Introduction

Selenium is an essential micronutrient for mammals, although even moderate doses are highly toxic. Selenium compounds act as an antioxidant or pro-oxidant, depending of the dose, chemical species and the nature of the target cell [1–4]. At nutritional levels, selenium exerts its biological activity through selenoproteins, which contain the amino acid selenocysteine [5]. A highly interesting feature of selenium is that tumor cells and especially highly resistant cancer cells are more sensitive to the cytotoxic effects of selenium as compared to benign and normal cells, thereby offering a therapeutic window and selenium is thus a highly interesting drug candidate for resistant cancer [2, 3]. Selenite reacts with extracellular thiols resulting in the production of the highly redox-active hydrogen selenide as intermediate metabolite [4, 6, 7]. In 2015, we published the first-in-man systematic phase I clinical trial for the use of iv selenium in cancer patients [8]. The results showed an unexpectedly high tolerance, short half-life, and fast clearance with minimum side effects below the maximal tolerable dose (MTD) at 10.2 mg/square meter body surface [8]. Taken together, selenite was proven to be a safe drug with favorable pharmacokinetic properties for repeated systemic use. Since then, several clinical trials have been published in particular for selenomethylselenocysteine (MSC) [9–11]. This organic selenium compound is naturally occurring in plants from selenium rich soils and this compound is not toxic and is thus a pathway for selenium detoxification in plants [2]. In mammals, however, MSC is metabolized by KYAT1 either through transamination [12, 13] to form methylselenopyruvate (MSP) or through β-elimination [14, 15] to form monomethylselenol (MMS). The latter metabolite is one of the most cytotoxic selenium compounds known and efficiently induces cell death, especially in rapidly dividing cells, indicating the great potential in the treatment of cancer [16–18]. MSP has also interesting properties in the context of cancer treatment since this compound has been shown to be an HDAC inhibitor and inhibits angiogenesis [12, 19]. The pharmacokinetic properties of MSC are very favorable with a short half-life, low inert toxicity and a high bioavailability for peroral use. In fact, MSC could best be described as a prodrug and the toxicity is decided by the activity and expression of KYAT1.

The presence of microRNAs (miRNA) has been known for 30 years and studying miRNAs has become a fast-growing area of research ever since [20, 21]. MiRNAs have been ascribed regulatory functions for gene expression and importance for expression of certain phenotypes as exemplified by miR-122, which is important for the phenotype of normal hepatocyte differentiation [22]. Generally, miRNAs are non-coding, small fragments of RNA, being remarkably stable compared to mRNA. Several studies suggest that circulating miRNA in serum could be a valuable tool for diagnostics and surveillance of disease progression of cancer and other diseases [23, 24]. For this reason, it is important to explore how different treatments affect the levels of miRNAs. Published data concerning pharmacological effects on the levels of miRNAs are very sparse and to our knowledge, no systematic investigations of the effects of treatment of tumor cells with selenium on microRNA expression have previously been performed. A possible effect of selenium on miRNA levels is expected since Alehagen et al., showed that the pattern of circulating miRNAs changed in a placebo-controlled supplement study, in which a cohort of elderly people was treated with a combination of nutritional levels of selenium along with Q10 [25].

Despite tremendous progress in the treatment and prognosis of some malignancies, including breast and prostate cancers where a great majority of patients is cured by current regimens, the prognosis remains very poor for patients with malignancies in visceral organs, in particular, cancers in the liver, bile ducts, and the pancreas [26]. These tumors are characterized by a pronounced inherent resistance to cytostatic drugs and novel therapeutic approaches including multikinase inhibitors and immunotherapy have so far resulted in disappointing results. This demonstrates a need for improved and different regimes that specifically target the characteristics of visceral malignancies. Several publications, by others, and us demonstrate outstanding efficacy of selenium in specifically killing highly resistant cancer cells [2, 3]. In order to develop selenium based treatment regimens, the effects of selenium on critical pathways in cancer cells must be explored. The miRNA pattern is in this context very important since some miRNAs have been proposed to determine the phenotype expressed by hepatocytes. The purpose of the present study was to explore any possible changes in the expression profiles of critical miRNAs in liver cancer cell lines and thus pave the way for future selenium based therapeutics.

## Materials and Methods

### Liver Cell Lines and Culture Conditions

Human hepatocellular carcinoma (HCC) cell lines HLE (RRID:CVCL_1281) and HLF (RRID:CVCL_2947), intrahepatic cholangiocarcinoma (CC) cell line HuH-28 (RRID:CVCL-2955) and extrahepatic CC cell line TFK-1 (RRID:CVCL_2214) were a kind gift provided by Stephanie Rössler (Institute of Pathology, Heidelberg University). HLE and HLF were maintained in DMEM (D6046, Sigma-Aldrich, St. Louis, MO, United States), HuH-28 and TFK-1 were cultured in RPMI 1640 (21875034, Life Technologies of Thermo Fisher Scientific Inc., Paisley, UK), supplemented with 10% fetal bovine serum (P40–37500, Pan-Biotech, Aidenbach, Germany), 100 U penicillin/0.1 mg streptomycin (P0781, Sigma-Aldrich, St. Louis, MO). RPMI 1640 was further supplemented with 2 mM L-Glutamine (G7513, Sigma-Aldrich, St. Louis, MO). Culturing was maintained at 37 °C in a humidified atmosphere containing 5% CO_2_.

### MSC and Sodium Selenite Treatment

Sodium selenite and MSC were supplied by Sigma-Aldrich (214485 and M6680, Sigma-Aldrich, St. Louis, MO). Both drugs were dissolved in double distilled water (50 mM for sodium selenite and 250 mM for MSC), then, aliquoted and kept at −20 °C.

For cell viability measurement, 5000 cells of HLE, HLF, HuH-28 and 8000 cells of TFK-1 were seeded in 96-well plates one day ahead of treatment. Regarding MSC, the applied concentrations ranged from 7.5 to 480 μM with doubling paces, except for TFK-1, where the final concentration was 1920 μM. The treatment concentrations for sodium selenite ranged from 0.625 μM to 40 μM with doubling paces for each cell line. The final treatment volume was 150 μl for HLE, HLF, HuH-28, and 200 μl for TFK-1.

Concerning the measurement of miRNA expression, 240,000 cells of HLE, HLF, HuH-28 and 400,000 cells of TFK-1 were seeded in 6-well plates in advance. Next day, HLE, HLF and HuH-28 cells were treated with 15, 30, 60 and 120 μM of MSC, whereas 240, 480 and 960 μM concentrations were further applied for TFK-1. Regarding sodium selenite, treatment concentrations of 2.5, 5, 10 and 40 μM were applied, except for HuH-28, which received 1.25, 2.5, 5 and 10 μM of the drug. The final treatment volume was 3 ml for each cell line.

Each treatment lasted for 72 h. The cell viability experiments were repeated 3 times and three biological replicates were applied in measuring miRNA expression.

### Cell Viability Assay

The inhibitory effect of MSC and sodium selenite on cell proliferation was measured by sulforhodamine B (SRB) assay. At 72 h following treatment, cell culture media was withdrawn and the cells were washed with 1xPBS. For fixation, the cells were treated with 70 μl of 10% trichloroacetic acid (TCA) for 1 h at 4 °C, rinsed five times with very gently running tap water and air-dried. Then, the cells were stained with 0.4% SRB (S1402, Sigma-Aldrich, St. Louis, MO), 1% acetic acid solution for 20 min at RT. Following the withdrawal of the stain, the cells were washed five times with 1% acetic acid and air-dried. Finally, the stain attached to cellular proteins of TCA-fixed cells was dissolved in 200 μl of 10 mM Tris-HCl (pH 8). The plates were stirred for 20–30 min and the color development was measured at 570 nm using an EL-800 microplate reader (BioTek Instruments, Winooski, VT). For each treatment, the data were normalized to the absorbance value of untreated cells.

### RNA Isolation and Measurement of miRNA Expression

At 72 h following treatment, the 6-well plates were placed on ice. Following removal of treatment culture media, the cells were washed with 1xPBS and RNA was isolated with TRIzol (Life Technologies of Thermo Fisher Scientific Inc., Carlsbad, CA) according to the instructions of the manufacturer. Briefly, the cells were lysed in 360 μl of TRIzol, collected in an Eppendorf tube and incubated for five min at RT. Following the addition of 72 μl of chloroform, the tubes were gently shaken by inversion for 15 s and incubated for three min at RT. The aqueous phase was separated by centrifugation at 12,000 x g for 15 min at 4 °C and removed into a new Eppendorf tube. When 180 μl of isopropanol had been added, the mixture was incubated for 10 min at 4 °C and centrifuged at 12,000 x g for 10 min at 4 °C. After the withdrawal of the fluid, the pellet was washed with 360 μl of 75% ethanol, vortexed briefly and centrifuged at 7500 x g for 5 min at 4 °C. Then all fluid was removed and the pellet was air-dried for 10 min. Finally, the pellet was dissolved in nuclease-free double distilled water. RNA concentration was quantified using an ND-1000 Spectrophotometer (NanoDrop Technologies Inc., Wilmington, DE). RNA samples were kept at −80 °C until further use.

We selected miRNAs that are abundantly expressed in normal liver (miR-21, -22, -24, -122, -125b, -143, -194, -199a, let-7a) according to Table 1 in Chen et al. [27]. Additionally, two further miRNAs related to cancer were selected, miR-210, involved in surviving hypoxia [28] and miR-224, promoting proliferation by AKT activation [29] as controls with hypothesized altered miRNA expression upon treatment. The expression of individual miRNAs was determined using the following TaqMan MicroRNA Assays (Life Technologies of Thermo Fisher Scientific Inc., Foster City, CA): miR-21-5p (ID: 000397), miR-22-3p (ID:000398), miR-24-3p (ID:000402), miR-miR-122-5p (ID:002245), miR-125b-5p (ID:000449), miR-143-3p (ID:002249), miR-194-5p (ID:000493), miR-199a-5p (ID:000498), miR-210-3p (ID:000512), miR-224-5p (ID:000599), let-7a-5p (ID:000377) and RNU48 (001006). Reverse transcription (RT) and quantitative polymerase chain reaction (qPCR) were performed according to the instructions of the manufacturer. Briefly, RT reaction was carried out using the TaqMan MicroRNA Reverse Transcription Kit (Life Technologies of Thermo Fisher Scientific Inc.) in a final volume of 7.5 μL containing 10 ng total RNA. The qPCR was performed using TaqMan Universal Master Mix II, no UNG (Life Technologies of Thermo Fisher Scientific Inc.) in a final volume of 10 μL containing 0.65 μL RT product. The amplification reaction was run in triplicates on a LightCycler 480 Instrument II (Roche Diagnostics, Indianapolis, IN). Relative expression was calculated by the 2^-ΔΔCq^ formula, applying RNU48 as the reference and normalized to the average ΔCq value of untreated cells. Fold change higher than 1.5 and lower than −1.5 (0.6) was regarded as an altered miRNA expression.

**Table 1:** Fold changes in HLE cell line treated with Se-methylselenocysteine and sodium selenite

**MSC (**μM**)**	**miR-21**	**miR-22**	**miR-24**	**miR-122**	**miR-125b**	**miR-143**	**miR-194**	**miR-199a**	**miR-210**	**miR-224**	**let-7a**
**Untreated**	1.0 (0.7–1.5)	1.0 (0.8–1.2)	1.0 (0.8–1.2)	1.0 (0.8–1.2)	1.0 (0.8–1.2)	1.0 (0.5–1.9)	1.0 (0.8–1.2)	1.0 (0.7–1.5)	1.0 (0.7–1.5)	1.0 (0.9–1.2)	1.0 (0.8–1.2)
**15**	1.3 (0.9–1.7)	**0.1** (0.1–3.5)	**0.1** (0.1–7.9)	1.4 (1.0–1.8)	0.9 (0.6–1.3)	0.9 (0.6–1.3)	**0.6** (0.4–1.0)	1.0 (0.5–2.0)	0.7 (0.5–0.8)	1.2 (0.9–1.5)	0.8 (0.6–1.0)
**30**	**2.2** (0.5–9.8)	0.8 (0.2–3.4)	1.1 (0.3–3.8)	**1.9** (0.8–4.2)	0.7 (0.2–2.2)	0.7 (0.2–2.1)	0.9 (0.2–3.8)	**2.2** (0.5–9.1)	0.9 (0.2–3.4)	1.0 (0.3–3.8)	0.9 (0.2–3.8)
**60**	0.8 (0.4–1.5)	1.2 (0.9–1.4)	1.1 (0.9–1.4)	1.3 (0.6–2.7)	1.0 (0.8–1.3)	0.7 (0.5–1.0)	0.8 (0.6–1.1)	**0.1** (0.1–1.8)	0.8 (0.6–1.0)	0.9 (0.6–1.3)	0.8 (0.6–1.0)
**120**	1.1 (0.4–2.6)	0.9 (0.7–1.1)	1.1 (0.8–1.7)	1.0 (0.6–1.7)	1.2 (1.1–1.3)	0.7 (0.4–1.4)	1.2 (0.8–1.9)	1.1 (0.8–1.5)	**0.6** (0.5–0.7)	1.2 (0.8–1.7)	0.9 (0.7–1.1)
**Se (μM)**	**miR-21**	**miR-22**	**miR-24**	**miR-122**	**miR-125b**	**miR-143**	**miR-194**	**miR-199a**	**miR-210**	**miR-224**	**let-7a**
**Untreated**	1.0 (0.5–2.1)	1.0 (0.8–1.3)	1.0 (0.6–1.6)	1.0 (0.7–1.5)	1.0 (0.8–1.3)	1.0 (0.4–2.5)	1.0 (0.6–1.6)	1.0 (0.7–1.4)	1.0 (0.6–1.6)	1.0 (0.6–1.8)	1.0 (0.6–1.7)
**2.5**	**1.6** (0.9–2.9)	1.0 (0.6–1.6)	1.3 (0.9–1.9)	**2.0** (0.8–4.9)	0.9 (0.5–1.7)	1.1 (0.7–1.7)	1.2 (0.8–1.9)	0.8 (0.4–1.7)	1.1 (0.7–1.6)	1.1 (0.7–1.8)	1.1 (0.7–1.6)
**5**	0.9 (0.3–2.3)	**0.6** (0.3–1.0)	1.0 (0.4–2.3)	1.2 (0.8–1.8)	**0.6** (0.5–0.9)	**0.4** (0.2–0.9)	1.0 (0.6–1.8)	0.8 (0.6–1.1)	0.7 (0.4–1.1)	0.8 (0.5–1.3)	0.7 (0.4–1.4)
**10**	1.2 (0.7–2.3)	**4.3** (2.2–8.4)	**3.6** (2.3–5.9)	**10.6** (6.3–17.8)	0.8 (0.4–1.4)	**3.2** (1.0–10.1)	**2.5** (1.4–4.5)	–	**1.5** (0.9–2.5)	**4.3** (2.5–7.6)	**0.4** (0.2–0.9)
**20**	**1.8** (1.0–3.1)	**14.5** (8.7–24.3)	**9.4** (5.1–17.3)	**84.8** (47.8–150.5)	**1.7** (1.0–2.8)	**17.3** (11.1–27.0)	**3.9** (0.9–16.6)	–	**3.9** (2.5–5.9)	**2.7** (0.6–12.1)	**0.3** (0.1–0.7)

MSC: Se-methylselenocysteine, Se: selenite, numbers in parenthesis: fold changes with SD, thick borderline represents the cut-off for IC_50_, −: undetectable

### Statistical Analysis

Results are expressed as mean ± SD. The analysis was performed by Student t-test or one-way ANOVA with 95% confidential interval followed by Tukey’s multiple comparison test (significant differences are indicated as *p < 0.05, **p < 0.01 & ***p < 0.001) compared with control and within the treatments. Statistical differences between IC_50_ values were determined by fitting nonlinear regression slopes on independent experiments (*n* ≥ 3). Data were analyzed with GraphPad Prism software, version 8.3.3 (538) (GraphPad Software Inc., San Diego, CA).

## Results

### Selenium Cytotoxicity in Hepatocellular Carcinoma and Cholangiocarcinoma Cell Lines

**Fig. 1:**
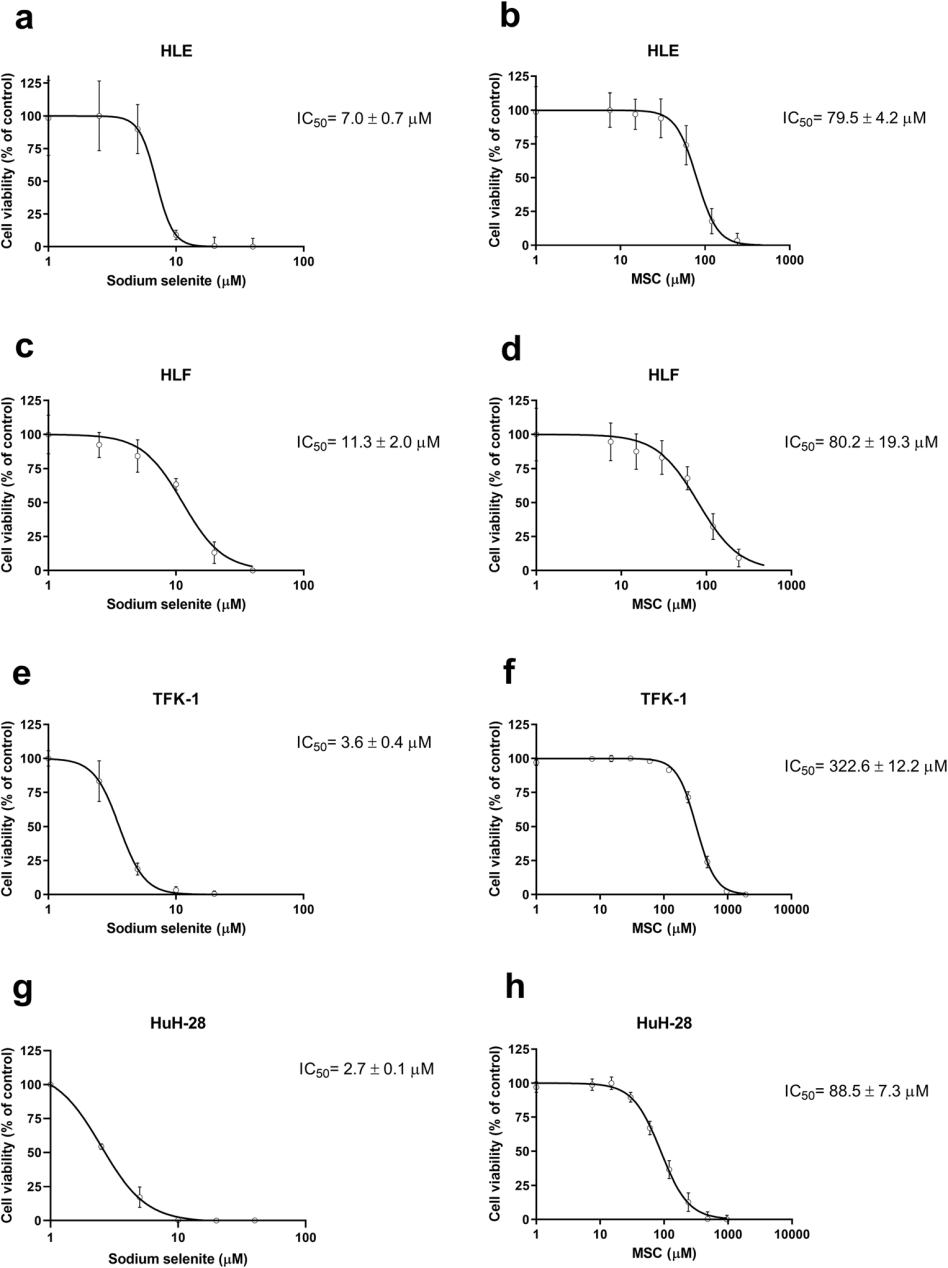
Selenium Cytotoxicity in Hepatocellular carcinoma and Cholangiocarcinoma cell lines. (**a**), (**c**), (**e**) & (**g**) Sodium selenite cytotoxicity in HLE, HLF, TFK-1, and HuH-28 cell lines. (**b**), (**d**), (**f**) & (**h**) Se-methylselenocysteine cytotoxicity in HLE, HLF, TFK-1, and HuH-28 cell lines. (**a**–**d**) Hepatocellular carcinoma and (**e**–**f**) Cholangiocarcinoma cell lines. IC_50_ is presented as an average of at least three measurements ± S.D.

In general, sodium selenite treatment resulted in much lower IC_50_ values compared to MSC in all the tested HCC and CC cell lines. In HLE cell line, the treatments resulted in an IC_50_ of 7.0 ± 0.7 μM for sodium selenite and 79.5 ± 4.2 μM for MSC (Fig. 1a–b). In HLF cell line, IC_50_ values of 11.3 ± 2.0 μM for sodium selenite and 80.2 ± 19.3 μM for MSC were found (Fig. 1c–d). In TFK-1 cell line, the IC_50_ values proved to be 3.6 ± 0.4 μM for sodium selenite and 322.6 ± 12.2 μM for MSC (Fig. 1e–f). In HuH-28, the treatments resulted in an IC_50_ value of 2.7 ± 0.1 μM for sodium selenite and 88.5 ± 7.3 μM for MSC (Fig. 1g–h).

### MiRNA Expression with Selenium Treatments

The miRNA patterns of the cell lines following MSC treatment differed from that observed following sodium selenite treatment, and each treatment led to differences in miRNA expression between the cell lines. During the miRNA measurements, low copy number of miR-122 and -199a in each cell line, of miR-143 in HLE, HLF, and TFK-1, and of miR-194 in HLE could be detected with Cq values around or above 35. The results are presented separately for each cell line.

### MiRNA Changes in Hepatocellular Carcinoma Cell Lines upon Selenium Treatments

**Fig. 2:**
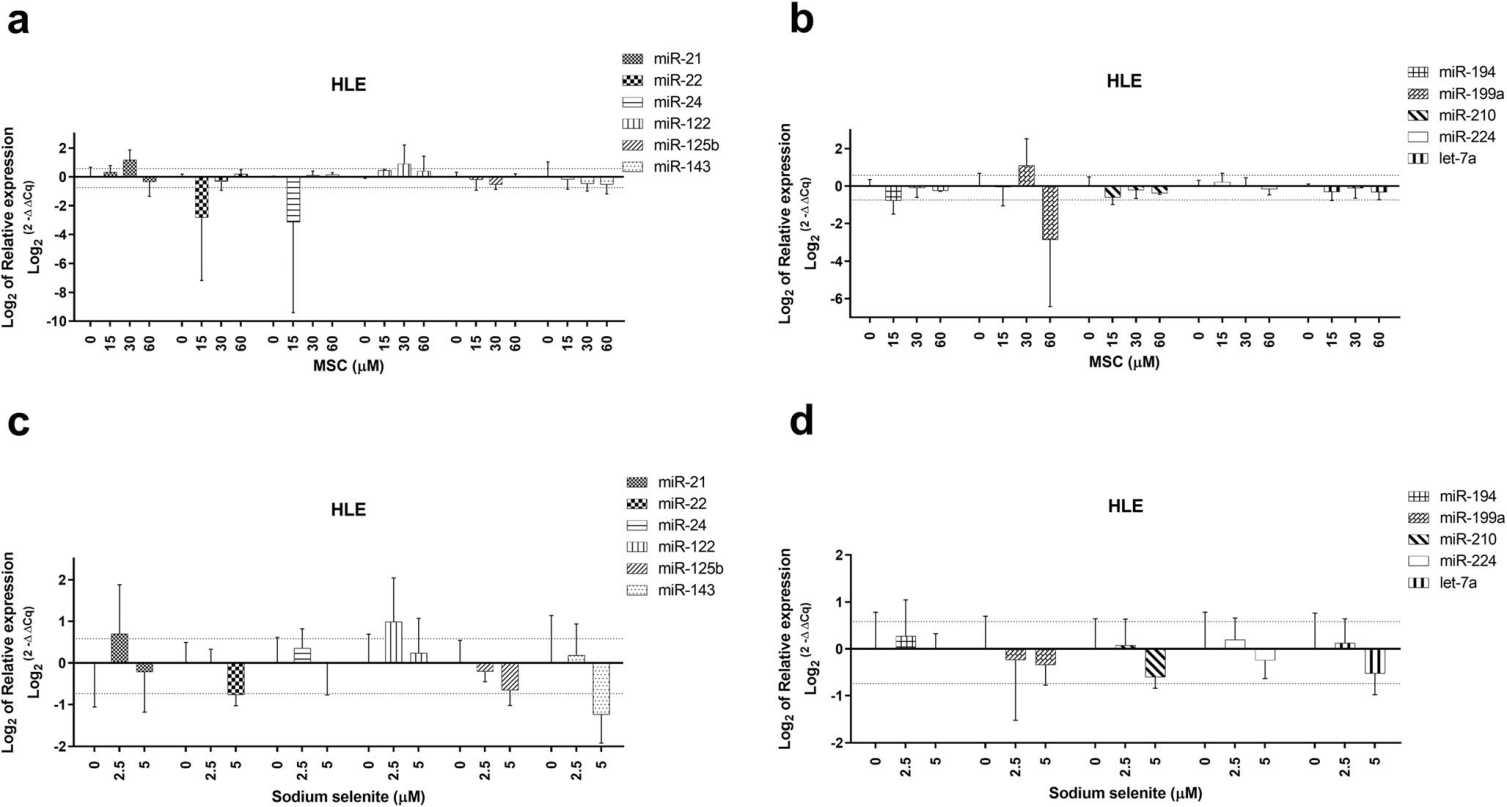
miRNA expression in HLE cells upon Se-methylselenocysteine and selenite treatment (**a**–**d**). miRNA expression patterns following MSC (**a**–**b**) and sodium selenite (**c**–**d**) treatment in the HLE cell line. Thin dotted lines signify the cut-off for 1.5 and -1.5 fold change compared to untreated cells (**a**–**d**). miRNA expression data shown are mean ± SD, statistical analysis performed with one-way ANOVA with 95% confidential interval followed by Tukey’s multiple comparison test (significant differences are indicated as *p < 0.05, **p < 0.01 & ***p < 0.001 compared with control and within the treatments)

In HLE cell line, the analyzed miRNAs showed no statistically significant alterations in their expression upon MSC or sodium selenite treatments (Fig. 2a–d), and only a few miRNAs exhibited fold change differences at concentrations below IC_50_ as compared to untreated cells. Namely, increased miR-21, -122, (for both drugs), -199a (for MSC) with fold changes between 1.6 and 2.2, and decreased miR-22 (for both drugs), -24, -194, -199a (for MSC), -125b, -143 (for sodium selenite) with fold changes from -1.6 to -10.0 were observed at individual concentrations below IC_50_ (Table 1). Regarding concentrations above IC_50_, MSC resulted in decreased levels of miR-210 (fold change -1.6), whereas sodium selenite treatment was associated with markedly increased miRNA expression (fold changes from 1.7 to 84.8); only let-7a was found to be decreased (fold change -2.5) (Table 1 – the formula to convert fold change below 1 provided in the Tables is -1/fold change).

**Fig. 3:**
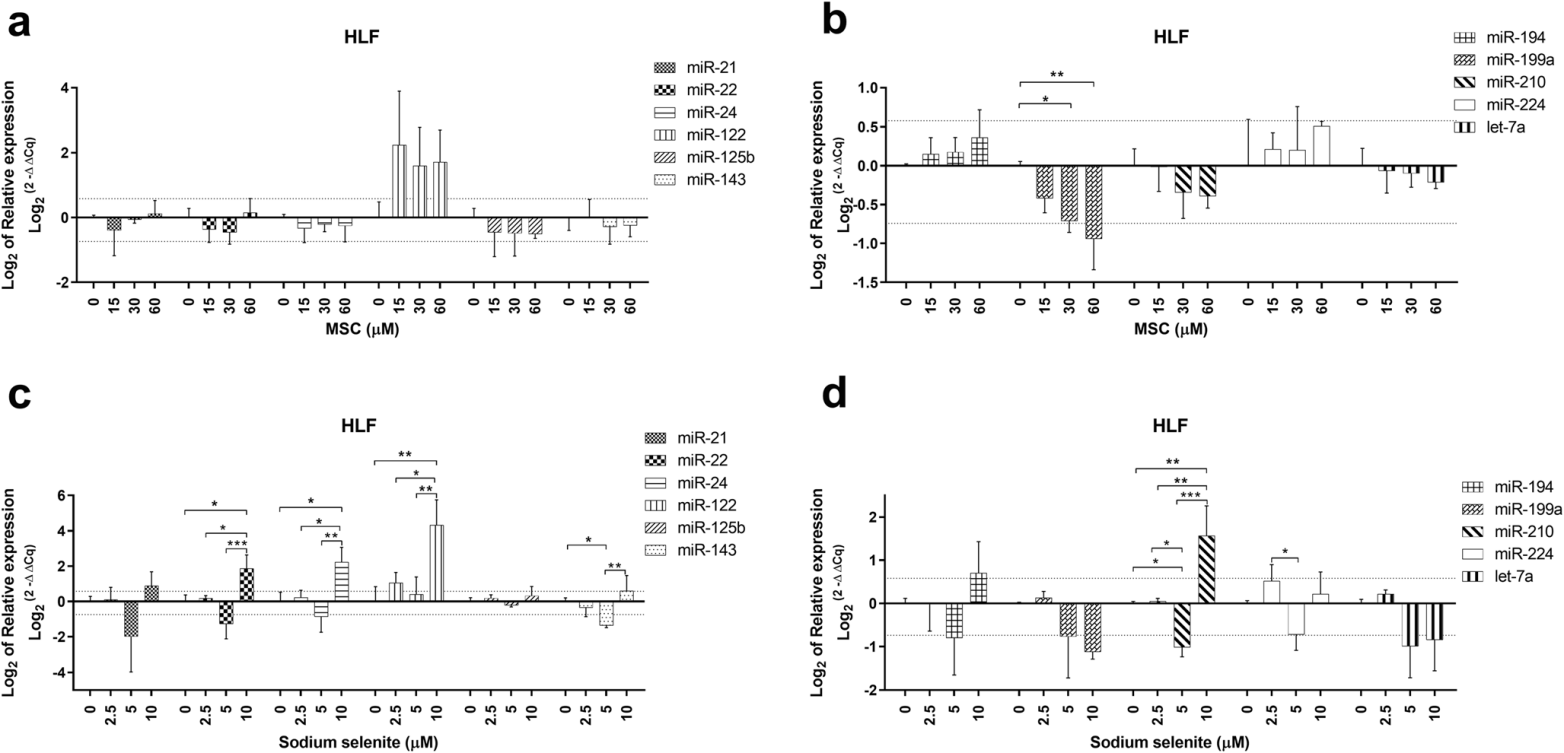
miRNA expression in HLF cells upon Se-methylselenocysteine and selenite treatment (**a**–**d**). miRNA expression patterns following MSC (**a**–**b**) and sodium selenite (**c**–**d**) treatment in the HLF cell line. Thin dotted lines signify the cut-off for 1.5 and  -1.5 fold change compared to untreated cells (**a**–**d**). miRNA expression data shown are mean ± SD, statistical analysis performed with one-way ANOVA with 95% confidential interval followed by Tukey’s multiple comparison test (significant differences are indicated as *p < 0.05, **p < 0.01 & ***p < 0.001 compared with control and within the treatments)

In HLF cell line, contrary to the IC_50_ values being similar to HLE, both drugs resulted in significantly altered miRNA expression at concentrations below IC_50_. For MSC, the levels of miR-199a were decreased at 30 and 60 μM (*p* < 0.05) compared to untreated cells (Fig. 3b). Based on fold change differences, miR-122 was increased at 15, 30 and 60 μM (fold changes between 3.0 and 4.7) and miR-199a was decreased at 30 and 60 μM (fold changes -1.6 and  -2.0) (Table 2). Regarding sodium selenite, increased miR-22, -24, -122, -210 at 10 μM (p < 0.05) and decreased miR-143, -210 at 5 μM (p < 0.05) were detected in comparison to untreated cells (Fig. 3c–d). Considering fold change differences, miR-21, -22, -24, -143, -194, -210 were decreased at 5 μM but increased at 10 μM (fold changes from -3.3 to 4.7), miR-122 was increased at 2.5 and 10 μM (fold changes 2.1 and 20.0) and miR-199a, -224, let-7a were decreased at 5 and/or 10 μM (fold changes between -1.6 and  -2.0) (Table 2). At concentrations above IC_50_, intensively increased miRNA expression was observed (fold changes from 1.9 to 55.8) with only let-7a found to decrease (fold change -1.6) upon sodium selenite treatment, whereas miR-122 increased (fold change 3.2) and miR-199a decreased (fold change -2.0) upon MSC treatment (Table 2).

**Table 2:** Fold changes in HLF cell line treated with Se-methylselenocysteine and sodium selenite

**MSC (**μM**)**	**miR-21**	**miR-22**	**miR-24**	**miR-122**	**miR-125b**	**miR-143**	**miR-194**	**miR-199a**	**miR-210**	**miR-224**	**let-7a**
**Untreated**	1.0 (0.9–1.1)	1.0 (0.8–1.2)	1.0 (0.9–1.1)	1.0 (0.7–1.4)	1.0 (0.8–1.2)	1.0 (0.8–1.3)	1.0 (1.0–1.0)	1.0 (0.9–1.1)	1.0 (0.9–1.2)	1.0 (0.7–1.5)	1.0 (0.9–1.2)
**15**	0.8 (0.4–1.4)	0.8 (0.5–1.2)	0.8 (0.6–1.1)	**4.7** (1.6–14.0)	0.7 (0.4–1.4)	1.0 (0.6–1.7)	1.1 (0.9–1.3)	0.7 (0.6–1.0)	1.0 (0.8–1.3)	1.2 (0.9–1.5)	1.0 (0.8–1.2)
**30**	1.0 (0.8–1.2)	0.7 (0.6–0.9)	0.9 (0.7–1.1)	**3.0** (1.3–6.9)	0.7 (0.5–1.1)	0.8 (0.6–1.1)	1.1 (0.9–1.4)	**0.6** (0.5–0.8)	0.8 (0.7–0.9)	1.2 (0.8–1.6)	0.9 (0.8–1.1)
**60**	1.1 (0.8–1.5)	1.1 (0.8–1.5)	0.8 (0.6–1.1)	**3.3** (1.7–6.3)	0.7 (0.7–0.8)	0.8 (0.7–1.0)	1.3 (1.0–1.7)	**0.5** (0.4–0.7)	0.8 (0.7–0.9)	1.4 (1.4–1.5)	0.9 (0.8–0.9)
**120**	0.9 (0.7–1.1)	0.9 (0.7–1.1)	1.0 (0.9–1.1)	**3.2** (1.4–7.5)	0.7 (0.6–0.8)	0.8 (0.5–1.2)	1.1 (0.9–1.3)	**0.5** (0.4–0.6)	0.8 (0.6–1.0)	1.1 (0.9–1.4)	0.7 (0.6–0.9)
**Se (μM)**	**miR-21**	**miR-22**	**miR-24**	**miR-122**	**miR-125b**	**miR-143**	**miR-194**	**miR-199a**	**miR-210**	**miR-224**	**let-7a**
**Untreated**	1.0 (0.9–1.2)	1.0 (0.8–1.3)	1.0 (0.7–1.4)	1.0 (0.5–1.9)	1.0 (0.9–1.2)	1.0 (0.8–1.3)	1.0 (0.8–1.2)	1.0 (0.8–1.2)	1.0 (0.8–1.2)	1.0 (0.8–1.2)	1.0 (0.9–1.2)
**2.5**	1.1 (0.7–1.6)	1.1 (0.8–1.6)	1.2 (0.9–1.5)	**2.1** (1.4–3.0)	1.1 (0.9–1.5)	0.8 (0.6–1.0)	1.0 (0.6–1.5)	1.1 (0.8–1.5)	1.0 (0.8–1.4)	1.4 (1.0–2.0)	1.2 (0.9–1.6)
**5**	**0.3** (0.1–1.3)	**0.4** (0.2–0.9)	**0.5** (0.2–1.3)	1.3 (0.5–3.2)	0.9 (0.6–1.1)	**0.4** (0.3–0.5)	**0.6** (0.3–1.3)	**0.6** (0.2–1.4)	**0.5** (0.3–0.7)	**0.6** (0.4–1.0)	**0.5** (0.2–1.1)
**10**	**1.8** (0.5–6.7)	**3.7** (1.0–13.1)	**4.7** (1.2–17.9)	**20.0** (9.6–42.0)	1.3 (0.4–3.9)	**1.5** (0.8–3.1)	**1.6** (0.5–5.8)	**0.5** (0.2–1.2)	**3.0** (0.8–10.4)	1.2 (0.4–3.7)	**0.6** (0.2–2.0)
**20**	**5.2** (2.3–11.9)	**18.0** (8.0–40.3)	**14.8** (6.1–36.2)	**55.8** (23.1–134.6)	**2.7** (1.2–6.2)	**15.7** (10.8–22.7)	**3.0** (1.3–7.0)	**2.6** (1.8–3.7)	**6.0** (2.5–14.2)	**1.9** (0.8–4.3)	**0.6** (0.2–1.9)

MSC: Se-methylselenocysteine, Se: selenite, numbers in parenthesis: fold changes with SD, thick borderline represents the cut-off for IC_50_

### MiRNA Changes in Cholangiocarcinoma Cell Lines upon Selenium Treatments

**Fig. 4:**
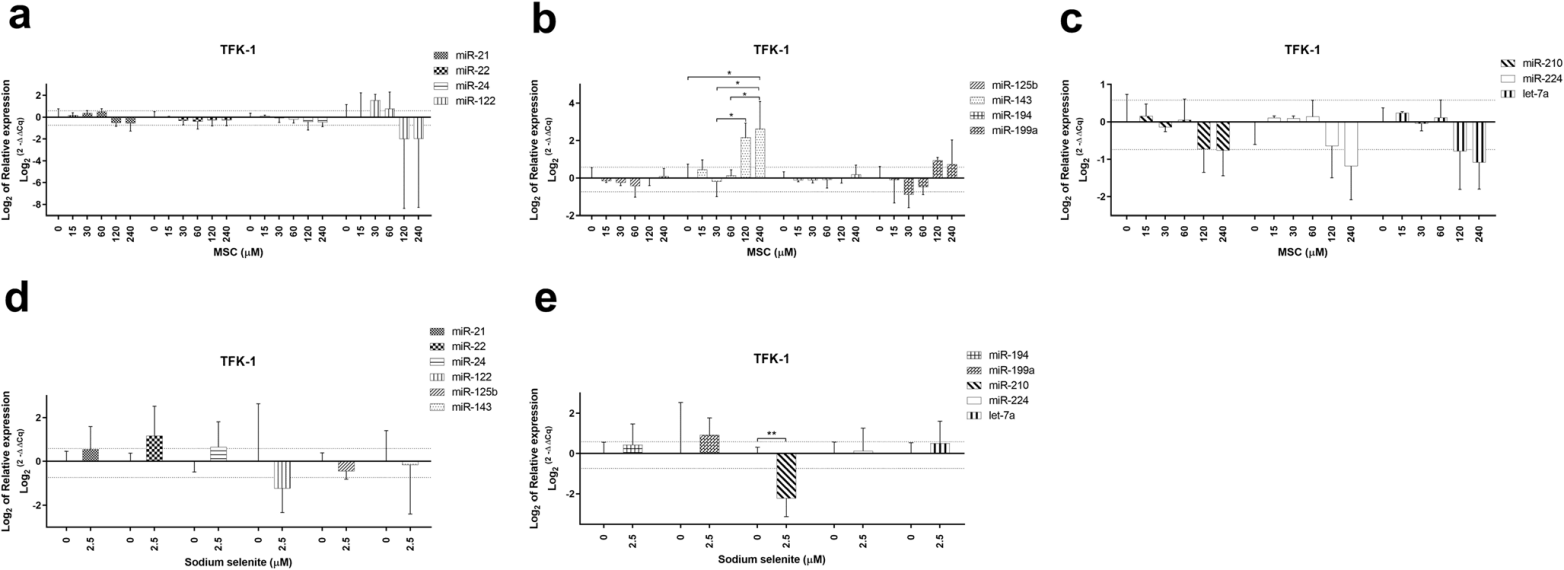
miRNA expression in TFK-1 cells upon Se-methylselenocysteine and selenite treatment (**a**–**e**). miRNA expression patterns following MSC (**a**–**c**) and sodium selenite (**d**–**e**) treatment in the TFK-1 cell line. Thin dotted lines signify the cut-off for 1.5 and  -1.5 fold change compared to untreated cells (**a**–**e**). miRNA expression data shown are mean ± SD, statistical analysis performed with one-way ANOVA with 95% confidential interval followed by Tukey’s multiple comparison test or Student t-test (significant differences are indicated as *p < 0.05, **p < 0.01 & ***p < 0.001 compared with control and within the treatments)

In TFK-1 cell line, the miRNA analysis upon MSC treatment revealed increased miR-143 at 240 μM (*p* < 0.05) when compared with untreated cells (Fig. 4b). Based on fold change differences, miR-122 increased at 30 and 60 μM (fold changes 2.9 and 1.7) but decreased at 120 and 240 μM (fold changes -1.6 and  -10), miR-143, -199a increased at 120 and 240 μM (fold changes from 1.7 to 6.2), whereas miR-199a, -210, -224 and let-7a decreased at 30, 120 and/or 240 μM (fold changes from -1.6 to -2.5) (Table 3.). As opposed to MSC, sodium selenite treatment resulted in decreased miR-210 (*p* < 0.01) at 2.5 μM compared to untreated cells (Fig. 4d–e). Regarding fold change differences, miR-22, -24, -199a were increased (fold changes between 1.6 and 2.6) and miR-122, -210 were decreased (fold changes -3.3 and  -8.3) at 2.5 μM (Table 3.). At concentrations above IC_50_, miR-143, -199a increased (fold changes between 2.4 and 5.7), miR-21, -22, -24, -210, -224 and let-7a decreased (fold changes from -1.6 to -5.0), and miR-122 increased and decreased (fold changes 3.3 and  -10.0) upon MSC treatment, whereas a markedly increased miRNA expression (fold changes between 2.0 and 8.5) were observed with only miR-122, -125b and  -210 decreasing (fold changes from -2.0 to -10.0) upon sodium selenite treatment (Table 3).

**Table 3:** Fold changes in TFK-1 cell line treated with Se-methylselenocysteine and sodium selenite

**MSC (**μM**)**	**miR-21**	**miR-22**	**miR-24**	**miR-122**	**miR-125b**	**miR-143**	**miR-194**	**miR-199a**	**miR-210**	**miR-224**	**let-7a**
**Untreated**	1.0 (0.7–1.5)	1.0 (0.8–1.3)	1.0 (0.8–1.3)	1.0 (0.4–2.4)	1.0 (0.8–1.3)	1.0 (0.7–1.5)	1.0 (0.8–1.3)	1.0 (0.7–1.4)	1.0 (0.7–1.4)	1.0 (0.7–1.3)	1.0 (0.8–1.3)
**15**	1.1 (1.0–1.3)	1.0 (1.0–1.1)	1.1 (1.0–1.2)	1.0 (0.2–4.8)	0.9 (0.8–0.9)	1.4 (0.9–2.0)	0.9 (0.8–1.0)	0.9 (0.4–2.1)	1.1 (0.8–1.5)	1.1 (1.0–1.2)	1.2 (1.1–1.3)
**30**	1.3 (1.1–1.5)	0.8 (0.6–1.0)	0.9 (0.8–1.2)	**2.9** (1.8–4.8)	0.8 (0.7–1.0)	0.9 (0.5–1.6)	0.9 (0.8–1.0)	**0.5** (0.3–0.9)	0.9 (0.8–1.0)	1.1 (0.9–1.2)	1.0 (0.9–1.1)
**60**	1.4 (1.2–1.7)	0.8 (0.5–1.1)	0.9 (0.7–1.1)	**1.7** (0.7–4.3)	0.7 (0.5–1.0)	1.1 (0.8–1.6)	0.9 (0.7–1.2)	0.7 (0.5–1.0)	1.0 (0.8–1.4)	1.1 (0.9–1.4)	1.1 (0.9–1.4)
**120**	0.7 (0.5–0.9)	0.8 (0.5–1.3)	0.7 (0.4–1.4)	**0.3** (0.0–18.5)	1.0 (0.6–1.5)	**4.5** (2.9–7.0)	1.0 (0.8–1.2)	**1.9** (1.3–2.8)	0.7 (0.4–0.9)	**0.6** (0.3–1.2)	**0.6** (0.3–1.2)
**240**	0.7 (0.4–1.0)	0.8 (0.6–1.2)	0.7 (0.5–1.0)	**0.1** (0.0–32.4)	1.1 (0.8–1.5)	**6.2** (1.9–20.4)	1.1 (0.8–1.6)	**1.7** (0.8–3.3)	**0.6** (0.4–0.8)	**0.4** (0.3–0.7)	**0.5** (0.3–0.7)
**480**	0.9 (0.3–3.3)	1.1 (0.7–1.8)	1.1 (0.5–2.2)	**3.3** (0.6–19.3)	1.1 (0.8–1.5)	**5.7** (4.2–7.6)	1.4 (0.6–3.2)	**4.0** (2.5–6.2)	0.7 (0.3–1.2)	**0.6** (0.4–0.9)	**0.6** (0.3–1.4)
**960**	**0.5** (0.1–3.1)	**0.6** (0.1–2.6)	**0.5** (0.1–2.6)	**0.1** (0.0–0.2)	0.8 (0.3–2.1)	**3.7** (1.6–8.8)	1.2 (0.3–4.5)	**2.4** (0.9–5.9)	**0.4** (0.1–1.3)	**0.3** (0.1–1.3)	**0.2** (0.0–1.3)
**Se (μM)**	**miR-21**	**miR-22**	**miR-24**	**miR-122**	**miR-125b**	**miR-143**	**miR-194**	**miR-199a**	**miR-210**	**miR-224**	**let-7a**
**Untreated**	1.0 (0.6–1.6)	1.0 (0.7–1.5)	1.0 (0.6–1.6)	1.0 (0.2–4.7)	1.0 (0.7–1.4)	1.0 (0.3–3.8)	1.0 (0.7–1.5)	1.0 (0.2–5.4)	1.0 (0.7–1.5)	1.0 (0.7–1.4)	1.0 (0.7–1.5)
**2.5**	1.4 (0.8–2.7)	**2.2** (1.0–5.1)	**1.6** (0.8–3.2)	**0.3** (0.2–0.3)	0.7 (0.5–1.0)	0.9 (0.2–3.5)	1.3 (0.7–2.5)	**2.6** (2.1–3.2)	**0.2** (0.1–0.5)	1.1 (0.5–2.2)	1.4 (0.7–2.9)
**5**	**4.3** (2.8–6.7)	**8.5** (5.0–14.3)	**4.0** (2.6–6.3)	**0.1** (0.0–0.5)	0.9 (0.5–1.5)	**3.8** (2.5–5.8)	**4.1** (2.6–6.4)	**3.5** (2.3–5.5)	**0.2** (0.1–0.2)	**2.2** (1.4–3.4)	**3.2** (2.1–5.1)
**10**	**4.4** (3.4–5.8)	**6.6** (5.1–8.6)	**3.5** (2.7–4.6)	**0.3** (0.1–0.7)	0.8 (0.6–1.0)	**3.7** (2.3–5.8)	**4.4** (3.1–6.2)	**3.9** (3.1–4.9)	**0.1** (0.1–0.1)	1.4 (1.1–1.9)	**2.3** (1.8–3.0)
**20**	**2.2** (1.0–5.0)	**5.1** (1.9–13.5)	**2.0** (0.8–5.0)	**0.4** (0.0–5.0)	**0.5** (0.2–1.5)	**5.1** (2.0–12.9)	**5.6** (2.2–14.1)	**5.8** (4.2–7.9)	**0.1** (0.0–0.2)	1.2 (0.6–2.6)	1.1 (0.3–3.6)

MSC: Se-methylselenocysteine, Se: selenite, numbers in parenthesis: fold changes with SD, thick borderline represents the cut-off for IC_50_

**Fig. 5:**
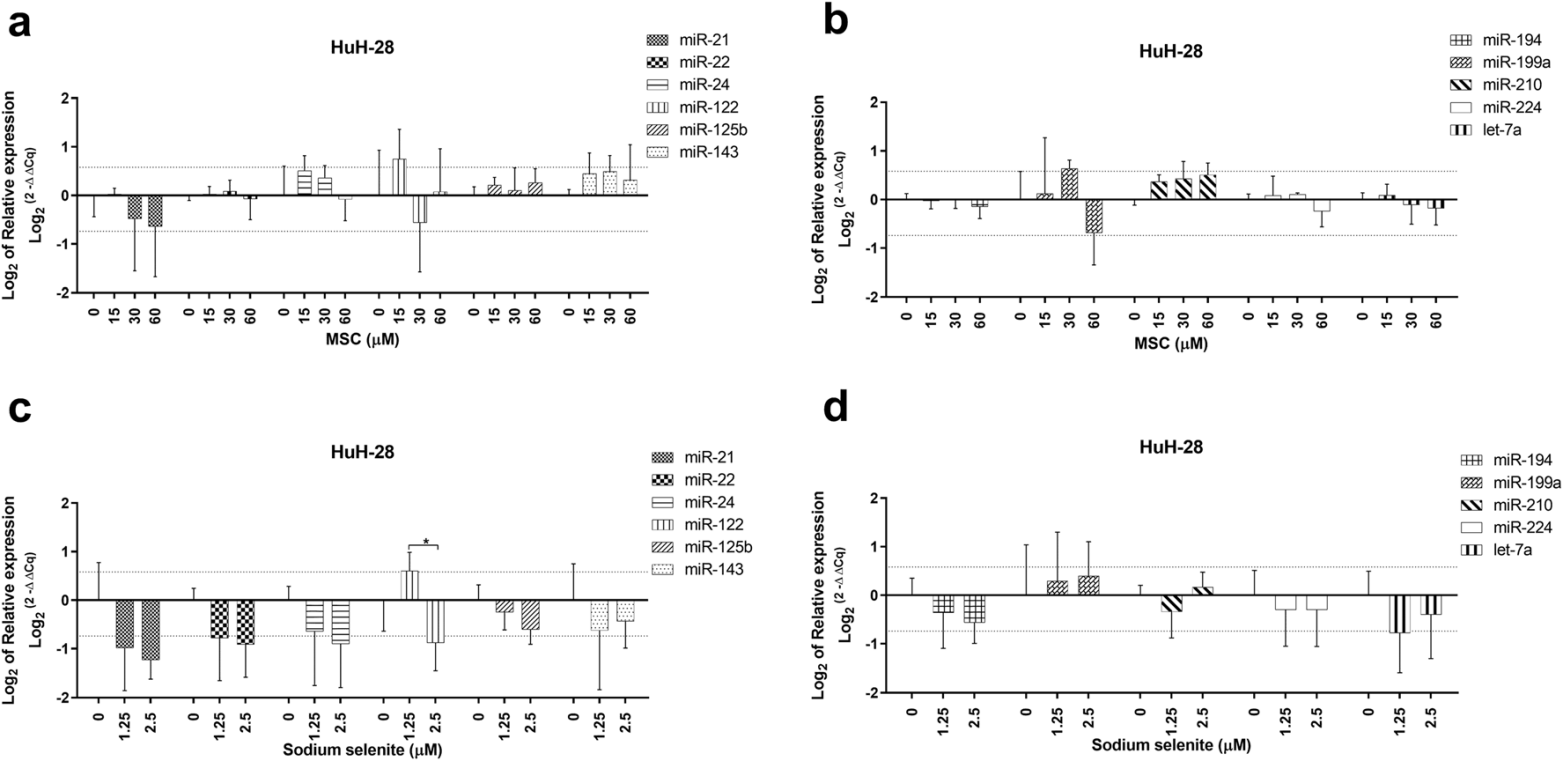
miRNA expression in HuH-28 cells upon Se-methylselenocysteine and selenite treatment (**a**–**d**). miRNA expression patterns following MSC (**a**–**b**) and sodium selenite (**c**–**d**) treatment in the HuH-28 cell line. Thin dotted lines signify the cut-off for 1.5 and  -1.5 fold change compared to untreated cells (**a**–**d**). miRNA expression data shown are mean ± SD, statistical analysis performed with one-way ANOVA with 95% confidential interval followed by Tukey’s multiple comparison test (significant differences are indicated as *p < 0.05, **p < 0.01 & ***p < 0.001 compared with control and within the treatments)

In HuH-28 cell line, no significantly altered miRNA expression was found in MSC or sodium selenite treated cells in comparison to untreated cells (Fig. 5a–d) and only a few miRNAs exhibited fold change differences at concentrations below IC_50_ compared to untreated cells (Table 4). Namely, miR-122 (for both drugs), -199a (only for MSC) were increased with fold changes between 1.5 and 1.7, and miR-21 (for both drugs), miR-199a (for MSC), -21, -22, -24, -122, -143, let-7a (for sodium selenite) were decreased with fold changes from -1.6 to -2.5 at individual concentrations below IC_50_ (Table 4). Regarding concentrations above IC_50_, sodium selenite treatment resulted in increased miR-22, -122, -194, -199a (fold changes between 1.5 and 8.3), and decreased miR-24, -125b, -224, let-7a (fold changes from -1.6 to -3.3), whereas MSC treatment led to decreased miR-21, -22, -24, -224 and let-7a with fold changes from -1.6 to -2.5 (Table 4).

**Table 4:** Fold changes in HuH-28 cell line treated with Se-methylselenocysteine and sodium selenite

**MSC (μM)**	**miR-21**	**miR-22**	**miR-24**	**miR-122**	**miR-125b**	**miR-143**	**miR-194**	**miR-199a**	**miR-210**	**miR-224**	**let-7a**
**Untreated**	1.0 (0.7–1.3)	1.0 (0.9–1.1)	1.0 (0.7–1.5)	1.0 (0.5–1.9)	1.0 (0.9–1.1)	1.0 (0.9–1.1)	1.0 (0.9–1.1)	1.0 (0.7–1.5)	1.0 (0.9–1.1)	1.0 (0.9–1.1)	1.0 (0.9–1.1)
**15**	1.0 (0.8–1.4)	1.0 (0.8–1.3)	1.4 (1.1–1.8)	**1.7** (1.2–2.3)	1.2 (0.9–1.5)	1.4 (1.0–1.8)	1.0 (0.7–1.3)	1.1 (0.5–2.5)	1.3 (1.0–1.7)	1.1 (0.8–1.4)	1.1 (0.8–1.3)
**30**	0.7 (0.3–1.5)	1.1 (0.9–1.2)	1.3 (1.1–1.5)	0.7 (0.3–1.4)	1.1 (0.8–1.5)	1.4 (1.1–1.8)	1.0 (0.9–1.1)	**1.6** (1.4–1.7)	1.3 (1.1–1.7)	1.1 (1.0–1.1)	0.9 (0.7–1.2)
**60**	**0.6** (0.3–1.5)	1.0 (0.6–1.4)	0.9 (0.6–1.4)	1.1 (0.5–2.3)	1.2 (0.9–1.6)	1.2 (0.7–2.2)	0.9 (0.7–1.2)	**0.6** (0.4–1.0)	1.4 (1.0–2.0)	0.8 (0.6–1.2)	0.9 (0.6–1.3)
**120**	**0.5** (0.2–1.2)	**0.5** (0.2–1.4)	**0.6** (0.2–1.7)	0.9 (0.1–5.6)	0.9 (0.5–1.7)	0.7 (0.3–1.7)	0.7 (0.3–1.7)	0.8 (0.6–1.2)	0.8 (0.4–2.0)	**0.5** (0.2–1.2)	**0.4** (0.2–1.1)
**Se (μM)**	**miR-21**	**miR-22**	**miR-24**	**miR-122**	**miR-125b**	**miR-143**	**miR-194**	**miR-199a**	**miR-210**	**miR-224**	**let-7a**
**Untreated**	1.0 (0.5–2.1)	1.0 (0.7–1.4)	1.0 (0.7–1.5)	1.0 (0.7–1.4)	1.0 (0.7–1.4)	1.0 (0.5–2.0)	1.0 (0.8–1.3)	1.0 (0.5–1.9)	1.0 (0.7–1.4)	1.0 (0.6–1.7)	1.0 (0.6–1.7)
**1.5**	**0.5** (0.2–1.0)	**0.6** (0.3–1.2)	**0.6** (0.3–1.5)	**1.5** (1.1–2.0)	0.8 (0.6–1.2)	**0.6** (0.2–1.7)	0.8 (0.5–1.3)	1.2 (0.6–2.5)	0.8 (0.5–1.3)	0.8 (0.4–1.6)	**0.6** (0.3–1.1)
**2.5**	**0.4** (0.3–0.6)	**0.5** (0.3–0.9)	**0.5** (0.3–1.0)	**0.5** (0.4–0.8)	0.7 (0.5–0.9)	0.7 (0.5–1.2)	0.7 (0.5–1.0)	1.3 (0.8–2.3)	1.1 (0.8–1.5)	0.8 (0.5–1.5)	0.8 (0.4–1.5)
**5**	1.2 (0.6–2.3)	0.7 (0.4–1.4)	**0.6** (0.3–1.0)	1.3 (0.8–2.1)	**0.6** (0.4–0.9)	1.4 (0.6–3.8)	1.1 (0.7–1.7)	**1.5** (1.0–2.3)	0.7 (0.4–1.1)	**0.3** (0.2–0.5)	**0.6** (0.3–1.2)
**10**	1.1 (0.5–2.4)	**1.6** (0.6–4.2)	1.0 (0.5–2.1)	**8.3** (5.5–12.5)	0.9 (0.4–1.8)	0.8 (0.4–1.6)	**2.9** (1.7–5.0)	**5.0** (2.3–10.9)	1.2 (0.6–2.5)	**0.3** (0.2–0.6)	**0.6** (0.3–1.6)

MSC: Se-methylselenocysteine, Se: selenite, numbers in parenthesis: fold changes with SD, thick borderline represents the cut-off for IC_50_

## Discussion

The purpose of the present study was to systematically explore the effects of the two leading selenium compounds in cancer research, selenite and MSC, on the expression of miRNAs, known to affect the differentiation and growth of tumor cells. Our data indicate rather sparse effects and the investigation was limited by the low basal levels of several miRNAs resulting in an expected high degree of inter-experimental variations thus making some results difficult to interpret.

Since long, there has been experimental evidence of chemo-preventive and chemotherapeutic properties of selenium compounds but it is not until lately these effects have been explored in clinical trials. The potential is great and we could expect the appearance of selenium-based therapeutic regimens in oncological treatment in a near future [2, 8].

The cytotoxicity of selenium is chemical species and cell type dependent [2, 3]. Especially drug-resistant cells are highly sensitive to the growth-inhibitory and cytotoxic effects of selenium, offering a therapeutic window for cancer treatment. Herein, we confirm the variable effects of selenium compounds on different cell lines. The MSC IC_50_ values were found to be similar in HLE, HLF and HuH-28 (around 80 μM), with each tumorous cell line originating form intrahepatic liver tumor, being HuH-28 an intrahepatic CC, whereas TFK-1 is an extrahepatic CC, which showed a much higher IC_50_ for MSC (322 μM). In contrast, the sodium selenite IC_50_ values were found to be similar in the two HCC cell lines (around 10 μM) and in the two CC cell lines (around 3 μM).

Recently the potential of miRNAs in diagnostics and cancer research has been recognized. MiRNAs can be detected in serum and may thus be a tool to follow disease progression and relapse [23, 24]. Furthermore, the regulatory properties of certain miRNAs may be used as drug targets or mediate drug effects. In the present investigation, we have focused on miRNAs possessing important roles for the normal function of the liver. Thus, these miRNAs are abundantly expressed in normal liver [27] and seven of them (miR-122, let-7a, miR-22, -125b, -143, -194 and  -24) are within the first 20 liver-specific miRNAs called “atlas liver” [30]. The expression levels of miR-122, let-7a, miR-22, -125b, -143, -194 and -199a have been described to be downregulated in liver cancer cells and function as tumor suppressor miRNAs, as these miRNAs are involved in inhibiting proliferation, cell cycle progression, epithelial-mesenchymal transition and activating apoptosis and autophagy [22, 31–38]. On the contrary, miR-21, -24, -210 and  -224 have been reported to be upregulated in HCC and function as oncomiRs, promoting proliferation, cell cycle progression, biliary tumor growth, angiogenesis and aggressiveness [31, 39–42]. In the present study, all miRNAs were found showing fold change differences in comparison to untreated state, however, only a few of these changes were statistically significant. Nevertheless, MSC treatment resulted in miRNAs showing less altered expression compared to selenite treatment.

Rather, cell line-dependent miRNA patterns could be observed. When comparing treated to untreated cells at concentrations below IC_50_, neither MSC nor sodium selenite treatment led to significantly altered miRNA expression in HLE and HuH-28 cell lines. MSC treatment, however, resulted in decreased miR-199a in HLF at 30 and 60 μM and increased miR-143 in TFK-1 at 240 μM; whereas sodium selenite treatment gave rise to altered levels of miR-210 in both HLF and TFK-1 (10 and 2.5 μM), and of miR-22, -24, -122 and  -143 in HLF (10 and 5 μM). This suggests that MSC, being a prodrug, brings about less alterations and, thereby, it may be less toxic to liver tumor cells as compared to selenite, which seems to affect the expression of miRNAs, regulating not only proliferation, apoptosis, EMT but also hypoxia (miR-210). In TFK-1, the adverse effect (decreasing oncomiR and increasing tumor suppressor miRNA) of sodium selenite on miR-210 and that of MSC on miR-143 seem to be beneficial considering the therapeutic use of selenium compounds. In association with fold change alterations, a predominantly increased miR-122 upon both treatments, an increased or decreased miR-199a upon MSC and miR-22 upon selenite treatment were observed in each cell line, indicating treatment specific alterations affecting miRNAs involved in regulating proliferation. The increase in the levels of miR-122 could also be regarded to be another beneficial effect of selenium compound treatment. Additionally, the adverse effects found upon selenium treatments were more beneficial in the case of the CC cell lines (indicated by decreased miR-21, increased miR-122 in HuH-28 and decreased miR-210 in TFK1 upon both treatments, decreased miR-24 in HuH-28 upon selenite, decreased miR-224 in TFK-1 upon MSC, increased miR-122, -143 in TFK-1 and increased miR-199a in HuH-28 upon MSC, and increased miR-22, miR-199a in TFK-1 upon selenite). Further, sodium selenite treatment resulted in altered miR-21, -143 in intrahepatic HLE, HLF, HuH-28, whereas MSC treatment led to altered miR-143, -210, -224 and let-7a in extrahepatic TFK-1, emphasizing further a cell line-dependent miRNA pattern following selenium treatment. Intriguingly, the levels of miR-125b showed no alteration with MSC in the cell lines and with selenite in HLF, TFK-1, and HuH-28. Further, no changes were observed in the levels of miR-143, -210, -224, let-7a in the HCC cell lines and in the levels of miR-22, -24, -194 in the CC cell lines upon MSC treatment, whereas miR-194, -224 were observed showing no alterations in the CC cell lines upon selenite treatment.

In conclusion, our results revealed that sodium selenite and MSC moderately altered miRNA expression in HCC and CC cell lines, resulting in not treatment- but rather cell line-associated miRNA expression patterns. Altogether, the most affected miRNAs were miR-122, -199a (being the first and third most highly expressed miRNAs in normal liver) for MSC and miR-122, -22 for sodium selenite. Further, miR-125b and  -194 seemed to be the most unaltered miRNAs upon treatment with both selenite and MSC.
